# The Nameless Knight

**Published:** 1886-03

**Authors:** 


### THE NAMELESS KNIGHT.

Dr. Merryman says he once witnessed a negro tournament, *a la mode* mediæval "chivalry." The herald—a burly darky—proclaimed the name of each Sir Knight as he entered the arena and took his turn. One fancifully clad and be-plumed darky, on a "fiery steed," was announced as the "Gallant Knight of the Seven Silver Moons ;" another was the "Knight of the Iron Mask ;" another the "Knight of the Golden "—something or other, and so on. Presently came an unknown and unnamed "Knight," a diminutive, queer-looking little cuss, as black as a coal, who looked like a monkey on horseback. As he entered, the herald asked for his title. The "Knight" whispered something, whereupon the herald shouted "Dis here one is de Knight of 'do-de-best-he-*kin*!'"

So, Dr. Merryman says our homœopathic "Knight" just "does the best he can."

In the journalistic field, adorned by such "Knights of the Quill" as Davis, Bell, H. C. Wood, Wile, Piffard, Connor, Matas & Bruns, Watson, Baldwin, Yandell, Miller, Eve, Westmoreland & Gray, Jaggard, Lanphear, Foster, Sim, Hays, Shoemaker, Edwards, Shrady, Brinton, Porter, and the peerless Marcy and others– all Princes of the Pen, our little friend on his homœopathic mustang cuts a sorry figure, with his sorry knowledge of the English language.

"W'ad some power the giftie gi' us--to see *ourselves*," etc.

